# Supplementary material for: Leaf-to-Whole Plant Spread Bioassay for Pepper and Ralstonia solanacearum Interaction Determines Inheritance of Resistance to Bacterial Wilt for Further Breeding
Source: Int J Mol Sci. 2021 Feb 25;22(5):2279. doi: 10.3390/ijms22052279 (PMC7956186; doi:10.3390/ijms22052279)
Supplement: Supplementary file 1 [file ijms-22-02279-s001.pdf]

Supplementary Table S1. Primer information used for RT-PCR analysis of cell death gene expression in this study.

Clone name	Sequence information (5'->3')		Tm (°C)	size (bp)	Accession No. (NCBI)	References
	Forward	Reverse				
CaHsr203J	CGCCATCAGCGTCTCCGTCTTC	CGCCCATCGGACATGTGATTG	58	413	AB162220	[34]
CaCDM1	AAAGGCGCAAACGGGAGCTG	TCACAGCCAAACGCTTCGATCC	60	400	GD056272	[53]
CaHIN1	ATCTTCACTATTCTCATCGTCCTTG	TGCAAACTGCTAGTATTCTTGTGAC	58	296	AB162221	[34]
CaPR10	ATGGGTGCTTATACCT	TTAAACATAGACAGAAGGAT	54	480	JF345171	[36]
CaPR4	ATCATGGAGAGTGTTACCAA	ATCCCAAGTAGCGCAGTAAG	58	171	AFN21550	[36]
CaAccOX	AGAAAGCTGCAGAGGAAAGCAAAC	TGAGATGCAACCGTTACTCCTATAC	58	343	XM_016698789	[36]
CaActin	CCACCTCTTCACTCTCTGCTCT	ACTAGGAAAAACAGCCCTTGGT	58	165	AY572427	[34]

Supplementary Table S2. Disease evaluation design and the number of plants to parents and their progenies based on disease severity index in 15, 20, and 30 dai against *R. solanacearum* SL1931 strain.

**Days after inoculation**	**Population [a]**	**No. of Plants**	**Disease severity index**					**Mean of DSI [b]**	**Wilt rate (%) [c]**	**AUDPC [d]**
			**0**	**1**	**2**	**3**	**4**			
15	MC4	30	8	22	0	0	0	0.7	0	7.5
	Subicho	30	0	0	0	0	30	4.0	100	50.3
	F_1	30	0	17	2	1	10	2.13	36.6	22.7
	F_2	90	0	60	3	7	20	1.89	32.2	21.9
20	MC4	30	6	24	0	0	0	0.8	0	7.5
	Subicho	30	0	0	0	0	30	4.0	100	50.3
	F_1	30	0	12	4	0	14	2.5	46.7	22.7
	F_2	90	0	44	11	1	34	2.3	38.8	21.9
30	MC4	30	0	30	0	0	0	1.0	0	16.5
	Subicho	30	0	0	0	0	30	4.0	100	90.3
	F_1	30	0	0	7	1	22	3.5	73.3	52.9
	F_2	90	0	1	41	3	46	3.0	54.4	48.5

[a] 'MC4' and 'Subicho' is resistance (R) and susceptible (S) parent line, respectively. The F_1 population crossed 'Subicho' (S) x 'MC4' (R) and F_2 population derived from self-cross of F_1 plants.

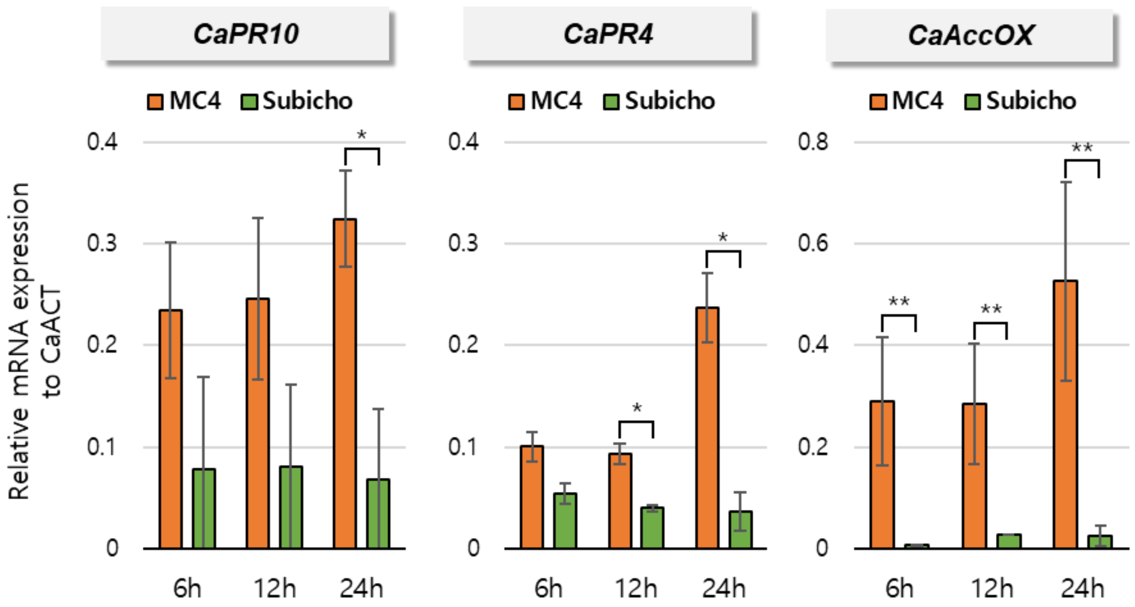

Supplementary Figure S1. Relative expression level of defense-related genes according to *CaPR1, CaPR4, and CaAccOX* was analyzed through *R. solanacearum* infection in 'MC4' and 'Subicho' leaves. Expression value was calculated and then normalized to *CaActin* expression. Asterisks indicate statistically significant differences according to Student's t-test (*$p < 0.05$, **$p < 0.01$).

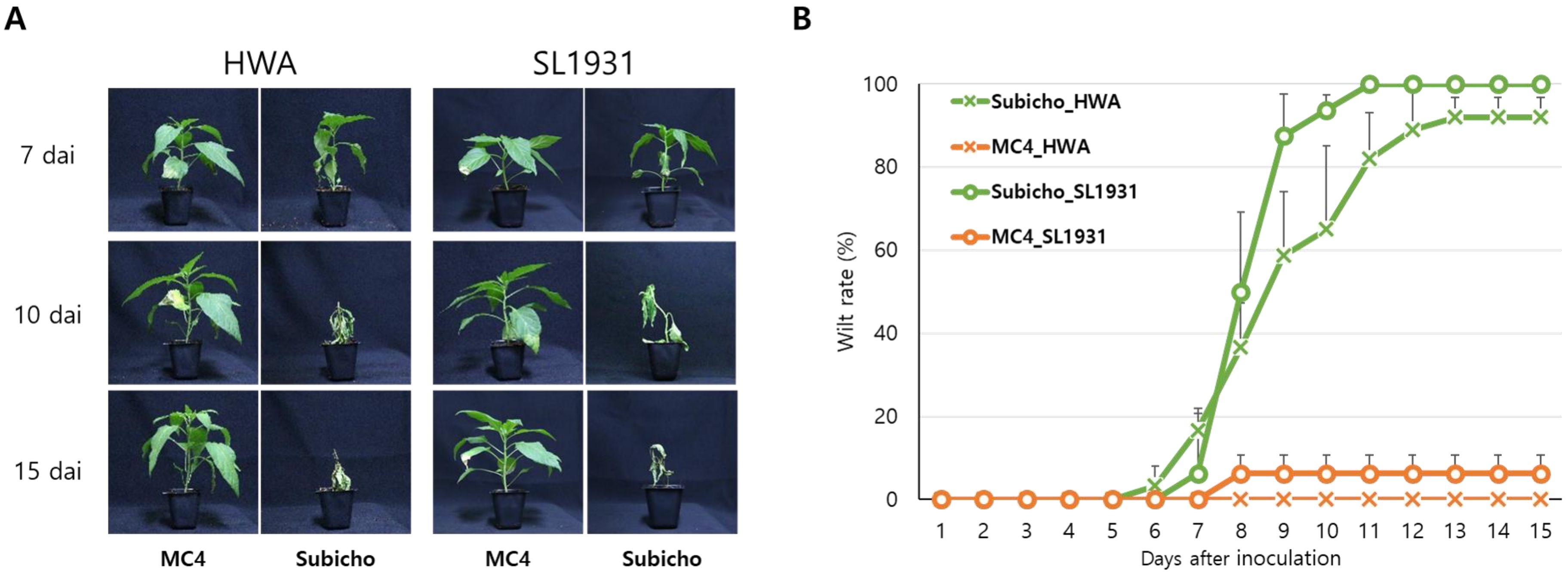

Supplementary Figure S2. Disease symptoms in ‘MC4’ and ‘Subicho’ with two *R. solanacearum* strains, SL1931 and HWA. (A), The phenotype of bacterial wilt (BW) in ‘MC4’ and ‘Subicho’ through leaf-inoculation with two *R. solanacearum* strains according at 7, 10 and 15 dai is shown. (B) Disease progression of BW disease on ‘MC4’ and ‘Subicho’. Disease severity of the plants was investigated every day after inoculation. In total, 30 plants were analyzed for each cultivar. Each data point represents the mean disease index for two independent experiment.
